# Age related clinical features of childhood Coeliac disease in Australia

**DOI:** 10.1186/1471-2431-5-11

**Published:** 2005-05-21

**Authors:** Monique L Stone, Timothy D Bohane, Kylie E Whitten, Vivienne H Tobias, Andrew S Day

**Affiliations:** 1 Departments of General Paediatrics, Sydney Children's Hospital, High St Randwick NSW 2031 Australia; 2 Gastroenterology, Sydney Children's Hospital, High St Randwick NSW 2031 Australia; 3 Nutrition & Dietetics, Sydney Children's Hospital, High St Randwick NSW 2031 Australia; 4 Department of Pathology, Anatomical Pathologist, Department of Patholgy, SEALS Sydney Children's Hospital, Randwick 2031 Australia; 5 School of Women's and Children's Health, University of New South Wales, Sydney, Australia

## Abstract

**Background:**

To describe the presenting clinical features of coeliac disease in a single paediatric centre, and to determine if the presenting features vary with age.

**Methods:**

A review was conducted of children who had been referred with clinical suspicion of coeliac disease to the paediatric gastroenterology department of a tertiary paediatric hospital in Sydney, Australia. Coeliac disease was defined using standard histological criteria. Medical records were reviewed retrospectively.

**Results:**

Clinical data were available for 74 cases of proven coeliac disease. Only 9% of patients were less than 2 years of age at diagnosis. Pre-school children (age <5 years) presented with different symptoms to school children (age ≥ 5 years). The most common presenting features in younger children were diarrhoea, irritability and weight loss. However, in older children, abdominal pain was the most common presenting feature.

**Conclusion:**

We found a significant difference in the clinical features of coeliac disease in pre-school compared to school age children.

## Background

There has been an apparent increase in the incidence of coeliac disease (coeliac disease) over the past 30 years [1]. For instance, the number of reported cases in the Netherlands increased from 0.18 per 1000 live births from 1975–1990, to 0.54 per 1000 live births 1993–1994 [1]. There is also increasing recognition that symptomatic coeliac disease may be the tip of the iceberg, with many more asymptomatic cases in the community [2-4].

It is unclear whether the changing prevalence of reported cases of coeliac disease is due to a real increase in the number of cases, enhanced awareness of disease or more reliable serological testing. Dietary changes, such as earlier introduction of gluten, have been suggested as contributing to the changing presentation of coeliac disease in some populations [5,6]. Breast feeding, in particular breast feeding after the introduction of gluten, is protective against the development of coeliac disease [7].

There are few data on the clinical features, incidence and prevalence of coeliac disease in Australian populations. In a population based screening study from a rural community of Western Australia, 10/3001 were positive for anti-endomysial antibodies. This community was of predominantly Anglo-Celtic origin [4]. Australians come from diverse ethnic backgrounds, thus the prevalence reported in this community cannot be generalised to the Australian population.

The primary aim of this study was to describe the clinical features of children diagnosed with coeliac disease at one tertiary paediatric centre in Australia. Our secondary aim was to determine the presentation patterns of coeliac disease in the local community at different ages.

## Methods

A retrospective cross sectional study was conducted at Sydney Children's Hospital, one of two tertiary referral paediatric hospitals in Sydney, Australia. The study population consisted of infants and children who had been referred to the Gastroenterology Department at Sydney Children's Hospital for investigation of possible coeliac disease between 1997 and 2002. Subjects were identified through records kept by the Departments of Nutrition and Dietetics, Gastroenterology, and Anatomical Pathology. Ethics approval was granted from the South East Sydney Area Health Service Research Ethics Committee-Eastern Section.

### Cases and clinical features

Cases were defined as those with histological confirmation of coeliac disease [8]. A single pathologist reviewed all slides. Where the histological diagnosis of coeliac disease was uncertain, clinical and laboratory features, including response to gluten free diet, were included.

The clinical details of each patient were extracted from the medical records retrospectively. Features of interest included presenting symptoms as well as the symptoms and signs elicited during consultation with a paediatric gastroenterologist. Weight and height measurements at diagnosis were documented. SD (standard deviation) scores were calculated using reference data [9]. The ethnic mix for the reference population of children admitted to Sydney Children's Hospital was described using child's country of birth.

### Laboratory investigations

The results of haemoglobin, iron studies, serum IgA, anti-gliadin (AGA) and anti-endomysial antibodies (EMA) were obtained from each patient's medical records. AGA and EMA tests had been performed at SEALS (South Eastern Area Laboratory Service, Kogarah, NSW). The SEALS laboratories measure EMA using indirect immunoflurescence, anti-human FITC conjugate IgA against monkey distal oesophagus. The slides are prepared by MeDICa (Encinitas, CA 92024). A value of 10 is used for as positive cut off. AGA were measured by commercial immunoassay kits (Analytica Ltd, Castle Hill NSW Australia). The cut off values used for children were IgA>25 and IgG >46.

Low haemoglobin values for age were defined as those less than the reference range for age used by the hospital laboratory (SEALS). The low end of the normal range for children 6 months to 2 years was 104 g/L; 2–4 years 107 g/L; 4–8 years 110 g/L; 8–12 y 113 g/L; >12 y 130 g/L. Patients were considered iron deficient on the basis of a microcytic blood film, low serum iron, low serum ferritin, and elevated iron binding capacity.

### Statistical analysis

Data were analysed by simple descriptive analysis performed manually and using Excel (Microsoft Office 97). Fisher's Exact tests were used to compare independent data using STATA [10]. Where data was not available, summary statistics were performed using only the data available.

## Results

### Subjects

**Figure 1:**
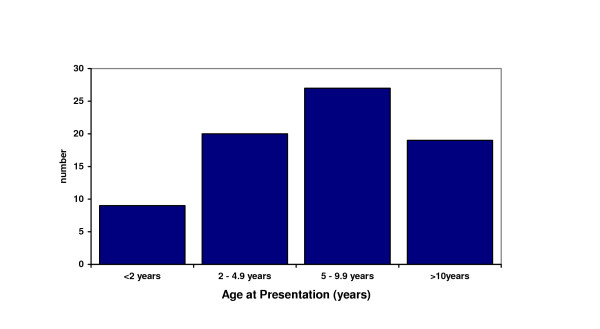
**Age at Presentation of Coeliac disease in the cohort of 74 subjects**. This figure illustrates the age distribution of the 74 subjects with coeliac disease in this study. Infants less than 2 years of age represented only 12% of the population. 27% of subjects were aged between 4 and 5 years, 36% were aged between 5 and 10 years, 26% were more than 10 years.

A total of 119 patients were identified over the six year period. Forty-three patients were excluded as there was another medical reason to explain their histological features or reason for dietetic referral. The medical records were unable to be located for two patients, leaving 74 subjects with coeliac disease available for analysis. The male: female ratio of patients was 1:2. The age at diagnosis ranged from 11 months to 14 years with median of 5.5 years. Seven patients (9% of total group) were less than 2 years of age (Figure 1). The ethnic mix (based on country of birth) of all admissions to SCH consists of approximately 94% Australian, 2% Asian, 1% UK and Europe, 0.05% Africa, and 0.02% Pacific Islands (data compiled by MS using admission data for Sydney Children's Hospital for 2002).

### Clinical features

Sixty nine of the seventy four children had presented with symptoms (Table 1). Full details of the presenting symptoms were available in 65 of the patients. Twenty nine of these children were aged less than five (preschool) and forty were aged five years or greater. The most common presenting symptoms in younger children (those <5 years) were diarrhoea (59%), irritability (34%) and weight loss (38%). In older children (≥ 5 years), the most common presenting feature was abdominal pain (55%), followed by diarrhoea (26%). Twenty-three percent of the symptomatic children had > 3 presenting symptoms.

**Table 1:** Clinical features of 69 symptomatic children with Coeliac disease

	**Age < 5 years n = 29 (% of group)**	**Age ≥ 5 years n = 40 (% of group)**
Abdominal pain	6 (20)	25 (62.5)*
Diarrhoea	17 (59)	12 (30)*
Tiredness/lassitude	9 (31)	9 (22.5)
Abdominal distension	16 (55)	9 (22.5)*
Constipation	4 (14)	5 (12.5)
Irritability	10 (34)	6 (15)*
Mouth Ulcers	2 (7)	6 (15)
Anaemia	5 (17)	10 (25)
Weight loss or lack of expected weight gain	11 (35)	6 (15)*
Anorexia and vomiting	7 (24)	0*

(*p < 0.05) This table displays the number of subjects with each symptom. The numbers in brackets describe the percentage of the age group with that symptom.

Five patients had no gastrointestinal symptoms at diagnosis. Four of these patients had positive coeliac serology during routine complication screening for type 1 diabetes (T1DM). The fifth patient had coeliac serology performed as part of investigation into recurrent infections.

Overall, ten children were noted to have other medical problems potentially related to the development of coeliac disease. Five patients had T1DM, two patients had Trisomy 21, two patients had juvenile rheumatoid arthritis, and one patient had Raynaud's phenomenon. Fourteen of the 79 (18%) patients had a family history of coeliac disease in a first degree relative.

The mean weights and heights of the subjects with coeliac disease were not significantly different to the reference sex and age matched population (mean weight SD -0.45 ± 0.14; mean height SD -0.4 ± 0.17). However, 5% of the children with coeliac disease had a weight SD score of more than 2 SD below the mean and 12% of children had a height SD score of more than 2 SD below the mean.

### Laboratory data

Seventeen of 64 (26%) patients with haemoglobin levels available had haemoglobin levels below the reference range for age (mean haemoglobin 118 g/L ± 0.2). Thirty two of 37 (86%) patients who had iron studies available were deemed iron deficient (low serum iron and ferritin). Of those who were biochemically iron deficient, 8 (25%) had normal haemoglobin values for their age.

IgG AGA was elevated in 97% of cases. IgA AGA was elevated in 94% of cases. There were no patients with IgA deficiency. EMA were elevated in 97% of this group of children. There were two patients with positive EMA antibodies who had negative AGA, on the other hand there was one patient (aged 9 years) with negative EMA who had positive AGA.

## Discussion

This study describes the clinical features at presentation in a group of Australian children diagnosed with coeliac disease during a time of increasing awareness of this disease and widespread availability of reliable laboratory screening tests. The children included in this retrospective review presented with a wide variety of clinical features. The most common clinical features of coeliac disease in younger children were the so-called classical symptoms of diarrhoea, irritability and weight loss. However, older children more commonly presented with abdominal pain. There were few cases without gastrointestinal symptoms in our cohort.

The clinical features of the children in this study were similar to the clinical features described from populations in New Zealand [11], South Yorkshire [12] and Sweden [13]. Although abdominal pain is a common symptom of older children with coeliac disease, there is no association between classical recurrent abdominal pain and coeliac disease [14].

Anaemia was identified in approximately one quarter of the cases. Furthermore, many children had laboratory evidence of iron deficiency but were not anaemic at the time of diagnosis. Unfortunately only 86% of subjects had a full blood count taken, and 50% had blood taken for iron studies. Thus the true prevalence of anaemia and iron deficiency is uncertain. Cross sectional studies have identified a high prevalence of coeliac disease in patients with iron or folate deficiency [15,16]. The incidence of coeliac disease in patients in the community presenting only with iron or folate deficiency has been reported at 4.7% [15]. Anaemia was reported in only 5% of children with coeliac disease from the Netherlands [1]. In the United Kingdom, iron deficiency anaemia was reported in approximately 20% of adults with coeliac disease. Although the current data may have underestimated the true incidence of anaemia and iron deficiency, it is likely higher than that reported in these previous cohorts.

Growth failure in height and weight was not as common in this cohort as was described in coeliac children from the Netherlands, where 49.7% of children had poor growth [1]. In our study, 5% of children had a weight more than 2 SD below the mean, and 12% of children had a height more than 2 SD below the mean. Growth failure is more commonly seen in children diagnosed at a younger age than in our study [17]. The discrepancy in reported growth failure may also be due to different definitions of growth failure. In addition, because this was a retrospective study, it was not possible to obtain adequate information about growth velocity or of the children's genetic growth potential.

The children in our study were older at the time of diagnosis than previous surveys of children with coeliac disease. In a cohort of patients diagnosed between 1950 and 1969, 70 of 91 were less than 2 years at the time of diagnosis and only 3 were more than 6 years. In a smaller cohort of children diagnosed between 1972 and 1975, 32 of 42 were less than 2 years at the time of diagnosis and 6 were greater than 6 years [18]. In the Netherlands, the proportion of children with coeliac disease aged < 2 yrs has remained at about 60% from 1975 to 1994 [1]. An epidemic in coeliac disease in Swedish Children in the 1980's paralleled the change in feeding practices at the time, in particular increased exposure to wheat, rye and barley and decreased duration of breast feeding [5]. A prospective study of coeliac disease in infants showed that a longer duration of breast feeding, smaller amounts of gluten in the diet in infants less than 12 months [17] and breast feeding after gluten is introduced to the diet reduces the risk of coeliac disease in infants less than 2 years [7]. Thus, the later age of acquiring coeliac disease in the Australian population may be due to the delayed introduction of solids in the diet of Australian children. The current recommendations in Australia are to introduce solids at 6 months (NHMRC Dietary Guidelines for Children and Adolescents in Australia 1993).

In this study, there were relatively few cases of coeliac disease detected by the screening of at risk groups. This contrasts with the worldwide trend of an increased prevalence of coeliac disease detected by screening [2,19-22]. A large multi-centered study of children and adults in the United States of America showed that the prevalence of celiac disease was 1:22 in first degree relatives, 1:56 in symptomatic individuals or a disorder known to be associated with celiac disease (type 1 diabetes, down syndrome, anemia, arthritis, osteoporosis, infertility and short stature), and 1:133 in the not-at-risk population [23].

In our study, the best screening test in children aged over 2 years with symptoms of, or who are at risk of, coeliac disease was IgA EMA. Other studies have shown that this test has a sensitivity of 95–98%, specificity of 94–95%, positive predictive value of 91–95% and negative predictive value of 96–98% [24]. For children less than 2 years of age, IgA AGA may be more reliable [24]. Increasingly over the last few years, reports suggest that tissue-transglutaminase (tTG) antibody may be even more reliable than EMA as a screening test [25,26], and in addition is more reproducible and reliable. This test was not available during the period of this review. Although the utility of these newer tests is improved compared to AGA antibodies, they are not yet accepted as diagnostic tests. Any child with symptoms suspicious of coeliac disease, even if negative serology, warrants further review by a paediatric gastroenterologist, as a small bowel biopsy remains the gold standard test for diagnosis of coeliac disease.

## Conclusion

Coeliac disease in children does not always present with classical clinical features, especially in older children. The possibility of coeliac disease should be considered in any child presenting with diarrhoea, irritability and weight loss, and particularly in older children with abdominal pain. With the widespread availability of minimally invasive screening tests for coeliac disease, identifying cases in at risk groups by screening should also be considered.

## Competing interests

The author(s) declare that they have no competing interests.

## Authors' contributions

MS conceived the study, performed the data collection and statistical analysis and drafted the manuscript; KW for assistance in collecting the data and reviewing the manuscript; VT performed the histological examination of the biopsy specimens; AD and TB were involved in the study design, writing and reviewing of the manuscript.

## Pre-publication history

The pre-publication history for this paper can be accessed here:



## References

[B1] K GE, Mearin ML, Franken HCM, Houwen RHJ, Hirasing RA, Vandenbrouche JP (1997). Twenty Years of childhood coeliac disease in The Netherlands: a rapidly increasing incidence?. Gut.

[B2] Day AS, Cook HB, Whitehead M, Abbott GD (2000). Anti-endomysial and anti-gliadin antibodies in screening for coeliac disease in children at greater risk of developing coeliac disease. N Z Med J JID - 0401067.

[B3] Csizmadia CGDS, Mearin ML, von Blomberg BME, Brand R, Verloove-vanhorick SP (2003). An iceberg of childhood coeliac disease in the Netherlands. The Lancet.

[B4] Hovell CJ, Collett JA, Vautier G, Cheng AJP, Sutanto E, Mallon DF, Olynyk JK, Cullen DJE (2001). High prevalence of coeliac disease in a population-based study from Western Australia: a case for screening?. Medical Journal of Australia.

[B5] Ivarsson A, Persson LA, Nystrom L, Ascher H, Cavell B, Dannaeus A, Lindberg T, Lindquist B, Stenhammar L, Hernell O (2000). Epidemic of coeliac disease in Swedish children. Acta Paediatrica.

[B6] Challacombe DN, Mecrow IK, Ellliott K, Clarke FJ, Wheeler EE (1998). Changing infant feeding practices and declining incidence of coeliac disease in West Somerset. Archives of Disease in Childhood.

[B7] Ivarsson A, Hernell O, Stenlund H, Persson LA (2002). Breast-feeding protects against celiac disease. American Journal of Clinical Nutrition.

[B8] Report of the Working Group of European Society of Paediatric Gastroenterology and Nutrition (2003). Revised Criteria for the Diagnosis of Coeliac Disease. Archives of Disease in Childhood.

[B9] Hamill PVV, Drizd TA, Johnson CL, Reed RB, Roche AF, Moore WM (1979). Physical Growth: National Centre for Health Statistics percentiles.. American Journal of Clinical Nutrition.

[B10] Corporation S (2003). Stata 6.0.

[B11] R U, ML Y, N S (1994). Coeliac Disease: incidence and Prevalence in Wellington 1985-1992. New Zealand Medical Journal.

[B12] Sanders DS, Hurlstone DP, Stokes RO, Rashid F, Milford-Ward A, Hadjivassiliou M, Lobo AJ (2001). Changing face of adult coeliac disease: experience of a single university hospital in South Yorkshire. Postgraduate Medical Journal.

[B13] Ludvigsson J, Ansved P, Falth-Magnusson K, Hammersjo J, Johansson C, Edvardsson S, al (2004). Symptoms and Signs have changed in Swedish Children with Coeliac disease. Journal of Pediatric Gastroenterology and Nutrition.

[B14] Fitzpatrick KP, Sherman PM, Ipp M, Saunders N, Macarthur C (2001). Screening for celiac disease in children with recurrent abdominal pain. Journal of Pediatric Gastroenterology and Nutrition.

[B15] Howard MR, Turnbull AJ, Morley P, Hollier P, Clarke A, Webb.R (2002). A prospective study on the prevalence of undiagnosed coeliac disease in laboratory defined iron and folate deficiency. Journal of Clinical Pathology.

[B16] Ransford RA, Hayes M, Palmer M, Hall MJ (2002). A controlled, prospective screening study of celiac disease presenting as iron deficiency anemia. Journal of Clinical Gastroenterology.

[B17] Ascher H, Holm K, Kristiansson B, Maki M (1993). Different features of coeliac disease in two neighbouring countries. Archives of Disease in Childhood.

[B18] Walker-Smith J, Murch S (1999). Diseases of the Small Intestine in Childhood.

[B19] Bingley PJ, Williams AJK, Norcross AJ, Unsworth DJ, Lock RJ, Ness AR, Jones RW (2004). Undiagnosed coeliac disease at age seven:population based prospective birth cohort study. British Medical Journal.

[B20] Carlsson AK, Axelsson IE, Borulf SK, Bredberg AC, Ivarsson SA (2001). Serological screening for celiac disease in healthy 2.5-year-old children in Sweden. Pediatrics JID - 0376422.

[B21] Bowron A, Moorghen M, Morgan JE, Osborne JR, Stansbie D, Stone JE (2000). Cost-effective strategy for the serological investigation of coeliac disease. Ann Clin Biochem JID - 0324055.

[B22] Chan AW, Butzner JD, McKenna R, Fritzler MJ (2001). Tissue transglutaminase enzyme-linked immunosorbent assay as a screening test for celiac disease in pediatric patients. Pediatrics JID - 0376422.

[B23] Fasano A, Berti I, Gerarduzzi T, Not T, Colletti RB, Drago S, Elitsur Y, Green PHR, Guandalini S, Hill ID, Pietzak M, Ventura A, Thorpe M, Kryszak D, Fornaroli F, Wasserman SS, Murray JA, Horvath K (2003). Prevalence of celiac disease in At-Risk and Not-at-Risk Groups in the United States. Archives of Internal Medicine.

[B24] Farrell RJ, Kelly CP (2002). Celiac Sprue. The New England Journal of Medicine.

[B25] Dickey W, McMillan SA, Hughes DF (2001). Sensitivity of serum tissue transglutaminase antibodies for endomysial antibody positive and negative coeliac disease. Scand J Gastroenterol JID - 0060105.

[B26] Salmaso C, Ocmant A, Pesce G, Altrinetti V, Montagna P, Descalzi D, Martino S, Bagnasco M, Mascart F (2001). Comparison of ELISA for tissue transglutaminase autoantibodies with antiendomysium antibodies in pediatric and adult patients with celiac disease. Allergy JID - 7804028.

